# Oral vitamin B_12 _therapy in the primary care setting: a qualitative and quantitative study of patient perspectives

**DOI:** 10.1186/1471-2296-6-8

**Published:** 2005-02-21

**Authors:** Jeff C Kwong, David Carr, Irfan A Dhalla, Denise Tom-Kun, Ross EG Upshur

**Affiliations:** 1 Department of Family and Community Medicine, University of Toronto, 263 McCaul Street, Toronto, Ontario, M5T 1W7, Canada; 2 Department of Public Health Sciences, University of Toronto, McMurrich Building, 12 Queen's Park Crescent W., Toronto, Ontario, M5S 1A8, Canada; 3 Department of Medicine, University of Toronto, Suite RFE 3-805, 190 Elizabeth Street, Toronto, Ontario, M5G 2C4, Canada; 4 Department of Sociology, York University, 2060 Vari Hall, 4700 Keele Street, Toronto, Ontario, M3J 1P3, Canada; 5 Primary Care Research Unit, Sunnybrook and Women's College Health Sciences Centre, 2075 Bayview Avenue, #E-349, Toronto, Ontario, M4N 3M5, Canada

## Abstract

**Background:**

Although oral replacement with high doses of vitamin B_12 _is both effective and safe for the treatment of B_12 _deficiency, little is known about patients' views concerning the acceptability and effectiveness of oral B_12_. We investigated patient perspectives on switching from injection to oral B_12 _therapy.

**Methods:**

This study involved a quantitative arm using questionnaires and a qualitative arm using semi-structured interviews, both to assess patient views on injection and oral therapy. Patients were also offered a six-month trial of oral B_12 _therapy. One hundred and thirty-three patients who receive regular B_12 _injections were included from three family practice units (two hospital-based academic clinics and one community health centre clinic) in Toronto.

**Results:**

Seventy-three percent (63/86) of respondents were willing to try oral B_12_. In a multivariate analysis, patient factors associated with a "willingness to switch" to oral B_12 _included being able to get to the clinic in less than 30 minutes (OR 9.3, 95% CI 2.2–40.0), and believing that frequent visits to the health care provider (OR 5.4, 95% CI 1.1–26.6) or the increased costs to the health care system (OR 16.7, 95% CI 1.5–184.2) were disadvantages of injection B_12_. Fifty-five patients attempted oral therapy and 52 patients returned the final questionnaire. Of those who tried oral therapy, 76% (39/51) were satisfied and 71% (39/55) wished to permanently switch. Factors associated with permanently switching to oral therapy included believing that the frequent visits to the health care provider (OR 35.4, 95% CI 2.9–432.7) and travel/parking costs (OR 8.7, 95% CI 1.2–65.3) were disadvantages of injection B_12_. Interview participants consistently cited convenience as an advantage of oral therapy.

**Conclusion:**

Switching patients from injection to oral B_12 _is both feasible and acceptable to patients. Oral B_12 _supplementation is well received largely due to increased convenience. Clinicians should offer oral B_12 _therapy to their patients who are currently receiving injections, and newly diagnosed B_12_-deficient patients who can tolerate and are compliant with oral medications should be offered oral supplementation.

## Background

Intramuscular injections of vitamin B_12 _(cobalamin) have been the mainstay of B_12 _deficiency treatment for decades. However, because approximately 1% of orally ingested B_12 _is absorbed via simple diffusion throughout the gastrointestinal tract (i.e., independently of intrinsic factor)[1,2], oral replacement with high doses of cobalamin is both effective and safe, regardless of the etiology of B_12 _deficiency [1,3-12].

Oral B_12 _therapy would decrease physician burden, increase patient control over therapy, and avoid patient discomfort and inconvenience. Switching patients from B_12 _injections to oral therapy would also result in savings to the health care system [13]. While some commentators have argued that clinicians should switch to oral B_12 _therapy [13-15], little is known about patients' views concerning the acceptability and effectiveness of oral B_12_.

Efforts to switch patients who are well established on parenteral therapy (and who have previously been told they require lifelong injections) may fail without an understanding of what factors influence patient acceptability of oral therapy. Therefore, we combined qualitative and quantitative methods to test the hypothesis that patients offered oral B_12 _are willing to switch to oral therapy and to explore the reasons for their choice.

## Methods

We administered a questionnaire to patients with B_12 _deficiency, offered the option of a six-month trial of oral replacement, and administered a follow-up questionnaire. Throughout the study, semi-structured interviews were conducted for the qualitative arm. We received ethics approval from the Sunnybrook and Women's College Health Sciences Centre Research Ethics Board. All patients provided informed consent.

### Quantitative arm

#### Data collection

**Figure 1:**
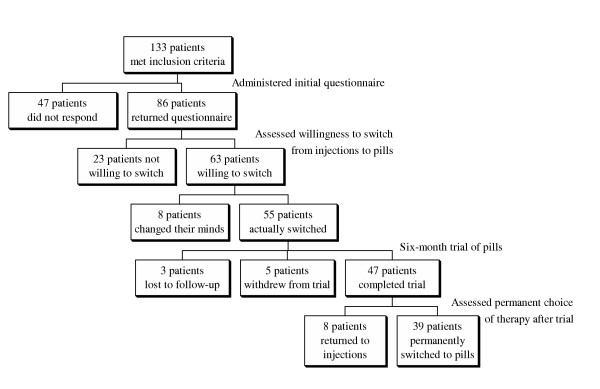
Flow diagram of patients in study.

The study took place at two academic family practice units and a community health centre with diverse practice profiles in Toronto. We included all patients who received regular injections (i.e., every 1 to 3 months), regardless of age and etiology of B_12 _deficiency. We excluded patients if their last injection occurred more than three months before the start of patient recruitment, if they had left the practice, if their B_12 _therapy had been discontinued or if they had already switched to oral therapy, if they did not speak English and did not have access to a translator, or if they had been recently diagnosed with a serious illness. After applying these criteria, 133 patients were included in the study (Figure 1).

The recruitment cover letter included one sentence describing the equivalency of oral B_12 _therapy to injections: "Studies have shown that vitamin B_12 _pills are just as effective and safe as B_12 _injections." Educational sessions on the effectiveness of oral therapy were held with the nursing staff (since they administer the injections) at all sites, and they were encouraged to pass this information on to their patients. As well, contact information for the study investigators was provided to the patients in case they had any questions.

We placed the initial questionnaire [see Additional file 1 ] in patients' charts to be completed at their next visit for a B_12 _injection. The initial questionnaire elicited demographic data, medication history, logistical aspects of B_12 _related visits, history of past B_12 _therapy, and attitudes about injection and oral therapy. Non-responders received up to two telephone reminders.

Patients willing to try oral therapy were given a six-month supply of B_12 _tablets (one 1000 μg tablet daily), and were offered testing of serum B_12 _levels at baseline (one month after their last injection) and after the six-month trial. Patients had the option to withdraw from the trial and return to injections at any time for any reason. A follow-up questionnaire was placed in the charts of patients who tried the pills. This questionnaire re-assessed patients' attitudes about the different forms of B_12 _therapy and asked whether they would continue on oral supplementation or return to injection therapy (see Additional file 2).

#### Statistical analyses

Where variables were not already dichotomous (e.g., for satisfaction with injections, the questionnaire listed "very satisfied," "satisfied," "neutral," "unsatisfied," and "very unsatisfied" as choices), we dichotomized the variable of interest (e.g., "satisfied" vs. "neutral/unsatisfied"). To ascertain the relationship between questionnaire responses and preference for oral therapy (as "willingness to switch" and "permanently switching") we performed bivariate and multivariate analyses. We used a p-value of 0.20 (for the continuity-adjusted χ^2 ^and Fisher's exact tests) as the cut-off for inclusion of individual patient factors into multivariate logistic regression models. We then performed backwards stepwise regression to determine statistically significant relationships after adjustment. We generated both crude and adjusted odds ratios with 95% confidence intervals.

### Qualitative arm

#### Participants and setting

One investigator (D.C.) conducted 17 semi-structured interviews in a private meeting room at the family practice unit. A purposive sample was selected amongst those willing to be interviewed to reflect diversity in terms of sex, age, willingness to switch, and final choice of therapy after the trial of B_12 _pills. These interviews were performed at various stages of the study (before, during, and after the trial of oral therapy). Amongst the 17 participants, four were selected from those not willing to switch, while of the 13 who were willing to try oral therapy, six were interviewed prior to, two during, and five after the trial of oral therapy.

#### Data collection and analysis

The interviews were audiotaped and transcribed. They lasted 20–30 minutes, with questions about the patient's knowledge and history of their B_12 _therapy, his or her relationship with health care providers, perceived advantages and disadvantages of both pills and injections, and attitudes about past and current B_12 _therapy. Analyses of the transcripts were performed independently by two investigators (J.K. and D.T-K.), using a three-step content analysis approach to identify and collate relevant themes [16]. Through a process of clarification, confrontation, and consensus, we reached agreement regarding the themes and sub-themes.

## Results

### Quantitative arm

For our initial questionnaire, we received responses from 86 out of the sample of 133 patients, for a response rate of 64.7%. Selected characteristics of the study population are presented in Table 1. Non-responders were younger and more likely to be female.

**Table 1:** Selected characteristics of study population and a comparison of responders vs. non-responders

	**Responders n = 86**	**Non-responders n = 47**	**p-value**
Mean age – Yr ± SD	72 ± 15	65 ± 20	0.03
Female sex – No. (%)	48 (56)	37 (79)	0.009
Level of education completed			
High school or less	28 (33)		
Some post-secondary education	21 (25)		
Bachelors degree or more	35 (42)		
Annual household income			
Less than $40K	40 (53)		
$40–79K	20 (26)		
$80K or greater	16 (21)		
Self-reported perception of health			
Above average	25 (29)		
Average	40 (47)		
Below average	20 (24)		
Prescription medications taken			
0	8 (10)		
1 to 3	46 (55)		
4 to 6	16 (19)		
7 +	14 (17)		
Monthly episodes of forgetting medications			
0	45 (54)		
1–2	26 (31)		
3+	12 (14)		
Years on B12 therapy			
0 to 2	19 (24)		
3 to 5	25 (31)		
6 to 10	17 (21)		
11 to 19	13 (16)		
20+	6 (8)		
Frequency of B12 injections			
Less than once monthly	9 (10)		
Once monthly	74 (86)		
More than once monthly	3 (3)		
Satisfaction with B12 injections			
Satisfied	22 (26)		
Neutral	59 (69)		
Unsatisfied	5 (6)		
Monthly visits to doctor for other reasons			
0	49 (60)		
1	23 (28)		
2 +	10 (12)		
Mode of travel to visit doctor			
Personal vehicle	49 (58)		
Public transit	23 (27)		
Walk	9 (11)		
Taxi	4 (5)		
Travel time to visit doctor			
< 15 min	27 (32)		
15–29 min	29 (35)		
30–44 min	20 (24)		
45–59 min	5 (6)		
60+ min	3 (3)		
Patients at each study site			
Sunnybrook Campus, SWCHSC*	51 (59)		
Flemingdon Health Centre	19 (22)		
Women's College Campus, SWCHSC*	16 (19)		

* Sunnybrook and Women's College Health Sciences Centre

#### Willingness to switch to oral B_12_

Sixty-three of our 86 respondents reported a willingness to switch to oral therapy. A large number of patient factors had no clear statistical relationship to a "willingness to switch" to oral therapy (i.e., p > 0.20) and were excluded from the multivariate model: age, education, income, drug insurance coverage, study site, number of prescription medications, mode of travel to the clinic, years of past B_12 _therapy, number of non-B_12 _related visits per month, improvement in perceived well-being since initiating B_12 _injections, several perceived disadvantages of injections (risk of complications, travel/parking costs), and several perceived disadvantages of pills (take too many pills already, would have to pay for them, won't work as well as injections).

The following factors were included in the multivariate model: gender, time to clinic, satisfaction with past B_12 _injections, several perceived disadvantages of injections (shots are painful, frequent visits to see MD/nurse, cost to health care system), and one perceived disadvantage of pills (won't see MD/nurse as often). Factors associated with willingness to switch after multivariate adjustment were: being able to get to the clinic more quickly (i.e., in less than 30 minutes) (OR 9.29, 95% CI 2.16–39.97), and believing that injection therapy is disadvantageous due to the need for frequent visits to health care provider (OR 5.41, 95% CI 1.10–26.56) and the increased costs to the health care system (OR 16.68, 95% CI 1.51–184.22) (Table 2).

**Table 2:** Patient factors associated with willingness to switch to oral therapy on initial questionnaire.

	**No. (%) of subjects**		**Unadjusted**		Adjusted*	
**Patient factor**	**Willing to switch n = 63**	**Not willing to switch n = 23**	**OR (95% CI)**	**p-value**	**OR (95% CI)**	**p-value**

**Time to clinic**						
0–29 minutes	49 (87)	7 (13)	9.34	<0.001	9.29	0.003
30+ minutes	12 (43)	16 (57)	(3.14–27.78)		(2.16–39.97)	
						
**Perceived disadvantages of injections**						
**Frequent visits to see MD/nurse**						
Agree	38 (86)	6 (14)	4.31	0.010	5.41	0.038
Disagree	25 (60)	17 (40)	(1.49–12.42)		(1.10–26.56)	
**Cost to health care system**						
Agree	26 (93)	2 (7)	7.38	0.010	16.68	0.023
Disagree	37 (64)	21 (36)	(1.59–34.23)		(1.51–184.22)	

* Adjusted for patient factors at least weakly associated (i.e., p < 0.20) with a willingness to switch to oral B_12 _therapy in bivariate analyses: gender, time to clinic, satisfaction with past B_12 _injections, perceived disadvantages of injections (shots are painful, frequent visits to see MD/nurse, cost to health care system), perceived disadvantage of pills (won't see MD/nurse as often)

#### Trial of oral therapy

Of the 63 patients who were willing to switch to oral B_12 _therapy, eight changed their minds before the trial started. Therefore, 55 patients were started on oral B_12_. Five dropped out and three were lost to follow-up, leaving 47 patients who completed the six-month trial. Reasons cited for discontinuing oral therapy included fatigue, neurological symptoms, and gastrointestinal intolerance.

Fifty-two patients returned the follow-up questionnaire. Over three-quarters (39 of 51 respondents) reported being satisfied or very satisfied with oral therapy. Only 8% of patients (4/51) perceived that they felt worse with pills, while 23.5% (12/51) felt better and the rest felt the same. Self-reported compliance was good; 48 patients or 92% reported forgetting to take the pills two times or less per month. Thirty-nine patients (71% of the 55 who actually switched) stated that they wished to permanently switch to B_12 _pills. Of the 35 patients who reported feeling the same with the pills as with injections, 28 (80%) chose pills.

Again, many patient factors had no association with a desire to permanently switch to oral B_12. _Patient factors that on bivariate analyses had a p-value of less than 0.20 and were consequently included in the multivariate model were: patient beliefs that frequent visits to see the doctor or nurse and the associated travel/parking costs are disadvantages of injections, and the belief oral B_12 _would add unnecessarily to an already large number of prescribed oral medications. Table 3 presents the factors significantly associated, after multivariate adjustment, with permanently switching to oral therapy: agreeing that disadvantages of injections included frequent visits to see the doctor/nurse (OR 35.41, 95% CI 2.90–432.70) and travel/parking costs (OR 8.66, 95% CI 1.15–65.30).

**Table 3:** Patient factors associated with permanently switching to oral therapy on follow-up questionnaire

	**No. (%) of subjects**		**Unadjusted**		Adjusted*	
**Patient factor**	**Choosing oral therapy n = 39**	**Choosing injection therapy n = 13**	**OR (95% CI)**	**p-value**	**OR (95% CI)**	**p-value**

**Perceived disadvantages of injections**						
**Frequent visits to see MD/nurse**						
Agree	27 (96)	1 (4)	27.00	<0.001	35.41	0.005
Disagree	12 (50)	12 (50)	(3.14–231.87)		(2.90–432.70)	
**Travel/parking costs**						
Agree	23 (92)	2 (8)	7.91	0.016	8.66	0.036
Disagree	16 (59)	11 (41)	(1.54–40.60)		(1.15–65.30)	

* Adjusted for patient factors at least weakly associated (i.e., p < 0.20) with permanently switching to oral B_12 _therapy in bivariate analyses: perceived disadvantages of injections (frequent visits to see MD/nurse, travel/parking costs), perceived disadvantage of pills (take too many pills already)

#### Serum B_12 _levels

**Figure 2:**
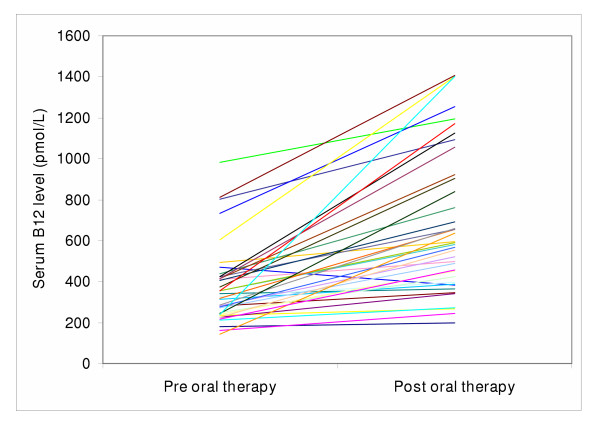
Serum B12 concentrations before and after six months of oral B12 therapy for forty patients.

Baseline and post-intervention serum B_12 _levels were obtained for 39 of the 55 patients who switched to pills (Figure 2). The mean serum level increased from 387 to 698 pmol/L (p < 0.0001) (reference range: ≥ 180 pmol/L, unlikely to have B_12 _deficiency). Only one patient's serum level decreased but even she remained within the normal range.

### Qualitative arm

The mean age of interview participants was 71 (range 54–92) years, with 7 men and 10 women.

#### Perceived efficacy of B_12 _therapy

Some patients felt that the B_12 _injections they had been receiving in the past were effective: "I find it gives me more energy." Patients who expressed this view were often not willing to switch to oral therapy, or if they did try the pills, they eventually went back to injections. Others felt that the injections hadn't helped much: "There is a little feeling that, 'Oh, I'm taking this. I should feel better,' but I'm not sure if I do." These patients tended to switch permanently to oral therapy.

When asked about the anticipated efficacy of oral therapy, some patients, particularly those who were not willing to switch, were sceptical: "Well, I don't think it will work. If the doctors suggested that I needed to go on the injections, I'm certainly pretty sure that the pills are not going to work. Otherwise he'd have put me on pills, would he not?" Many others were unsure about the efficacy of oral therapy.

#### Reasons for switching to oral therapy

Nearly every patient cited convenience as an advantage of switching to B_12 _pill. Other advantages of oral therapy included savings to the health care system and ease of travel. Cited disadvantages of injections included decreased compliance due to the need for frequent visits and potential complications associated with injections. A number of patients decided to switch because they were interested in participating in a research study. Some patients acknowledged that oral therapy would benefit those who are averse to needles, however, needles did not bother most of the patients interviewed: "...needles don't bother me, because – well, I don't know. They just don't seem to bother me. I know some people are very concerned about it."

#### Satisfaction with oral therapy

For both patients interviewed during their trial of oral B_12 _and for three of the five patients interviewed afterward, all of whom permanently switched to oral therapy, there was a high level of satisfaction with oral B_12_. One patient even reported feeling better on oral B_12_: "Well, I'm more level. I don't feel at the end of the month that I'm running out of energy. I'm quite well aware of that." (see Additional file 3).

#### Reasons for staying with/switching back to injection therapy

Disadvantages of switching to oral therapy included having to take an additional oral medication, fear of side effects, concern about swallowing difficulties, the inconvenience of taking a medication daily, and the potential for losing contact with health care providers and the opportunity for minor drop-in consultations. Some patients were under the impression that injections would work more quickly and directly.

Two patients who switched back to injections after trying the pills were interviewed. These patients switched back due to side effects – one cited neurological and gastrointestinal symptoms (her post-trial serum B_12 _level was significantly increased from baseline) and the other cited decreased energy (his post-trial serum B_12 _level was not available). (see Additional file 4).

## Discussion

We found that switching patients from injection to oral B_12 _is both feasible and acceptable to patients. Of those who responded to our initial questionnaire, nearly three-quarters were willing to try oral B_12_, and of those who did switch, most were satisfied and the majority wished to remain permanently on oral therapy. These patients believed that injections are disadvantageous because they are associated with too many visits to their health care provider and with higher patient costs (in terms of travel and parking expenses). In the qualitative arm of our study, most patients cited convenience as the reason for wanting to switch from injections to pills. We were also able to confirm findings from other studies indicating that oral B_12 _is biochemically equivalent to, if not better than, parenteral therapy.

One non-intuitive finding from the initial questionnaire assessing the factors associated with trying oral therapy (Table 2) was that those who were able to get to the clinic more quickly (i.e., in fewer than 30 minutes) were more likely to try switching. While one might expect that those who live closer to their clinic would benefit less from the convenience of not having to visit their health care provider as frequently, we speculate that perhaps these patients are more comfortable with trying oral therapy because they know that it is easy for them to access care if they need it, rather than having to rely on regular visits for their injections to see their primary care provider. Another interesting finding – that the perception of travel expenses being a disadvantage of the injections was not associated with being willing to try injections but was associated with permanently switching after the trial – suggests that perhaps patients did not realize the benefit of the saved travel costs until several visits had been averted.

By monitoring trough serum B_12 _levels (i.e., 1 month after their last injection) and again 6 months after oral therapy, we observed that one patient was a non-responder (i.e., serum level decreased), and 4 patients had serum levels that increased but remained below 295 pmol/L (a level considered by some to be borderline B_12 _deficient)[17]. This represents 13% out of the 39 patients who underwent testing, suggesting that follow-up testing may be warranted with serum B_12 _levels and, if available, functional tests such as serum homocysteine and methlymalonic acid. The sub-optimal response in these patients may be due to patient non-compliance or sub-optimal dosing (we used a 1-mg daily dose, rather than the 2-mg daily dose used by Kuzminski *et al*.) [8]. Further studies may be required to clarify the optimal dosing regimen for oral therapy.

The primary strength of our study was that we achieved triangulation through the use of both quantitative and qualitative methods. By using questionnaires, we were able to provide estimates such as switch rates and levels of patient satisfaction; with the qualitative arm, we were able to more thoroughly explore patient perspectives.

The main limitation of our study was the relatively small sample size for the quantitative arm, which led to large confidence intervals for the estimates of the relationship between patient factors and switching to oral B_12_. However, the sample was large enough to identify a number of patient factors associated with switching from injection to oral therapy and to determine that patient attitudes toward oral B_12 _therapy are greatly influenced by its convenience. A second limitation was that we did not assess the knowledge and attitudes of our patients' physicians and nurses, as they may have influenced patient's expectations and perceptions regarding the effective of oral B_12 _therapy. Finally, another limitation was the potential participant bias, evidenced by the differences between responders and non-responders in terms of age and sex. However, among the responders, neither age nor sex was significantly associated with switching to oral therapy, suggesting that any differences between participants and non-participants were likely irrelevant.

While there have been several studies examining physicians' perspectives on switching from intramuscular to oral therapy [18-21], we know of few studies that have examined patients' views. In a recent study that involved switching forty patients to oral therapy, the authors reported that 83% preferred the oral form [15]. However, no further details on patient perspectives were reported. Another study found that patients become very attached to receiving their injections; after identifying 48 patients who did not meet diagnostic criteria for B_12 _deficiency and providing educational sessions to encourage discontinuing injections, 38% stated they would leave the practice if denied their injections [22]. Explanations suggested by the authors include the reluctance to discontinue a therapy initiated by a trusted and respected physician, and the belief that intramuscular injections are more potent and more effective than medications taken orally. While the perception of increased potency of injections was reported by some patients in the qualitative arm of our study, this belief was supported by only 20% of respondents in the initial questionnaire (data not shown).

While oral B_12 _therapy may be acceptable for most patients, it may not be appropriate for those who will not be compliant with oral medications, such as patients with significant memory impairment or cognitive dysfunction, unless they have caregivers who could ensure compliance. Oral therapy also may not be appropriate for those with swallowing difficulties; for such patients, sublingual B_12 _therapy, which has been shown to be equally effective, may more appropriate [23].

## Conclusion

In summary, the results of our study suggest that clinicians should offer oral B_12 _therapy to their patients who are currently receiving injections if they can tolerate and are compliant with oral medications. Most patients who switch from parenteral to oral therapy are satisfied and wish to stay permanently on oral therapy. They often cite the inconvenience and the travel-related expenses associated with frequent visits to the doctor as disadvantages of parenteral therapy. Therefore, oral B_12 _therapy is generally well-received by patients, and should be considered by clinicians as a superior alternative to the traditional injections for most patients.

## Competing interests

The author(s) declare that they have no competing interests.

## Authors' contributions

JK designed and coordinated the study, analyzed the qualitative and quantitative data, and drafted the manuscript. DC conducted the in-depth interviews and assisted in data collection. ID helped to develop the questionnaire and performed statistical analyses of the quantitative data. DTK analyzed the qualitative data. RU conceived the study and provided guidance on all aspects of the project. All authors participated in the development of the manuscript and gave approval to its final submission for publication.

## Pre-publication history

The pre-publication history for this paper can be accessed here:



## Supplementary Material

Additional File 1 Initial questionnaire Click here for file

Additional File 2 Follow-up questionnaire Click here for file

Additional File 3 Reasons for switching to oral therapy Click here for file

Additional File 4 Reasons for not switching to oral therapy Click here for file

## References

[B1] Berlin H, Berlin R, Brante G (1968). Oral treatment of pernicious anemia with high doses of vitamin B12 without intrinsic factor. Acta Med Scand.

[B2] Schjonsby H (1989). Vitamin B12 absorption and malabsorption. Gut.

[B3] Waife SO, Jansen CJ, Crabtree RE, Grinnan EL, Fouts PJ (1963). Oral vitamin B12 without intrinsic factor in the treatment of pernicious anemia. Ann Intern Med.

[B4] Reisner EH, Weiner L, Schittone MT, Henck EA (1955). Oral treatment of pernicious anemia with vitamin B12 without intrinsic factor. N Engl J Med.

[B5] Crosby WH (1980). Oral cyanocobalamin without intrinsic factor for pernicious anemia. Arch Intern Med.

[B6] McIntyre PA, Hahn R, Masters JM, Krevans JR (1960). Treatment of pernicious anemia with oral administered cyanocobalamin (vitamin B12). Arch Intern Med.

[B7] Ungley CC (1950). Absorption of vitamin B12 in pernicious anemia: oral administration without a source of intrinsic factor. BMJ.

[B8] Kuzminski AM, Del Giacco EJ, Allen R, Stabler SP, Lindenbaum J (1998). Effective treatment of cobalamin deficiency with oral cobalamin. Blood.

[B9] Bolaman Z, Kadikoylu G, Yukselen V, Yavasoglu I, Barutca S, Senturk T (2003). Oral versus intramuscular cobalamin treatment in megaloblastic anemia: a single-center, prospective, randomized, open-label study. Clinical Therapeutics.

[B10] Andres E, Kurtz JE, Perrin AE, Maloisel F, Demangeat C, Goichot B, Schlienger JL (2001). Oral cobalamin therapy for the treatment of patients with food-cobalamin malabsorption. American Journal of Medicine.

[B11] Andres E, Kaltenbach G, Noel E, Noblet-Dick M, Perrin AE, Vogel T, Schlienger JL, Berthel M, Blickle JF (2003). Efficacy of short-term oral cobalamin therapy for the treatment of cobalamin deficiencies related to food-cobalamin malabsorption: a study of 30 patients. Clinical & Laboratory Haematology.

[B12] Roth M, Orija I (2004). Oral vitamin B12 therapy in vitamin B12 deficiency. American Journal of Medicine.

[B13] van Walraven CG, Austin P, Naylor CD (2001). Vitamin B12 injections versus oral supplements. How much money could be saved by switching from injections to pills?. Canadian Family Physician.

[B14] Lederle FA (1991). Oral cobalamin for pernicious anemia: medicine's best kept secret?. JAMA.

[B15] Nyholm E, Turpin P, Swain D, Cunningham B, Daly S, Nightingale P, Fegan C (2003). Oral vitamin B12 can change our practice. Postgrad Med J.

[B16] Miller WL, Crabtree BF (1994). Qualitative analysis: how to begin making sense. Family Practice Research Journal.

[B17] Snow CF (1999). Laboratory diagnosis of Vitamin B12 and folate deficiency. Arch Intern Med.

[B18] Lokk J, Nilsson M, Norberg B, Hultdin J, Sandstrom H, Westman G (2001). Vitamin B12 in primary health care and geriatrics – attitudes, knowledge and competence. International Journal of Geriatric Psychiatry.

[B19] Lokk J, Nilsson M, Norberg B, Hultdin J, Sandstrom H, Westman G (2001). Shifts in B12 opinions in primary health care of Sweden. Scandinavian Journal of Public Health.

[B20] Lokk J, Nilsson M, Norberg B, Rudolphi O, Sandstrom H, Westman G (1997). Controversies around vitamin B12 in Sweden. Attitudes and values behind clinical decision-making in primary health care. Hematology.

[B21] Nilsson M, Lokk J, Norberg B, Hultdin J, Sandstrom H, Westman G (2002). Sex differences in cobalamin vitamin B12 opinions of Swedish physicians. Nordic Journal of Psychiatry.

[B22] Lawhorne L, Ringdahl D (1989). Cyanocobalamin injections for patients without documented deficiency. JAMA.

[B23] Delpre G, Stark P, Yaron N (1999). Sublingual therapy for cobalamin deficiency as an alternative to oral and parenteral cobalamin supplementation. Lancet.

