# Supplementary material for: Oral vitamin B12 therapy in the primary care setting: a qualitative and quantitative study of patient perspectives
Source: BMC Fam Pract. 2005 Feb 21;6:8. doi: 10.1186/1471-2296-6-8 (PMC554115; doi:10.1186/1471-2296-6-8)
Supplement: Additional File 1 — Initial questionnaire [file 1471-2296-6-8-S1.pdf]

Initial questionnaire

1. Name: ___________________________

2. Phone Number (Day): ______________________

3. Phone Number (Evening): ______________________

4. Date of Birth: (mm/dd/yyyy) _____________________

5. Gender: □ M □ F

6. Marital Status:
   □ Single □ Married □ Divorced □ Separated □ Widowed □ Other

7. Highest level of education completed: *(please check one)*
   □ Did not complete high school □ Bachelor's degree
   □ High school □ Graduate or Professional degree
   □ Some post-secondary education

8. Household income in the past year, before taxes and deductions:
   □ < $20,000 □ $60,000 – 79,999 □ $150,000
   □ $20,000 – 39,999 □ $80,000 – 99,9999
   □ $40,000 – 59,999 □ $100,000 – 149,999

9. Personal perception of health:
   □ Poor □ Below average □ Average □ Above average □ Excellent

10. *If you are 65 years of age or OLDER, please skip this question.* If you are UNDER the age of 65, do you have an insurance plan that pays for prescription medications?
    □ Yes □ No

11. How many different types of prescription medications do you take? ____

12. How many different types of over-the-counter medications or vitamins do you take by mouth? ____

13. On average, how many times in a month do you forget to take your medications?
    □ 0 □ 1-2 □ 3-5 □ 6-9 □ 10+

14. How do you get to the hospital to visit your doctor most of the time? *(please select only ONE)*
    □ I walk □ I drive □ Somebody else drives me
    □ Taxi □ Bus/Subway □ Wheel-Trans
    □ Other (please specify) ________________________

15. How long does it normally take you to get to the hospital to visit your doctor?
 <15 minutes 30-44 minutes 60 minutes
 15-29 minutes 45-59 minutes

16. How many years have you been receiving B_{12} therapy?
 0-2 yrs 3-5 yrs 6-10 yrs 11-19 yrs 20 yrs

17. How often do you get your B_{12} injections?
 More than once per month
 Once per month
 Less than once per month

18. In a typical month, how many times do you see your doctor for reasons other than getting your B_{12} injection?
 0 1 2 3-4 5+

19. Have B_{12} injections made you feel better?
 Definitely not Probably not Maybe Probably Definitely

20. How satisfied are you with your B_{12} treatment?
 Very unsatisfied Unsatisfied Neutral Satisfied Very satisfied

21. Do you think B_{12} pills will be as effective as B_{12} injections?
 Definitely not Probably not Maybe Probably Definitely
 Don't know

22. Has your doctor ever mentioned taking B_{12} pills instead of injections to you?
 Yes No

23. What do you feel are the disadvantages of getting B_{12} by injection? *(select all that apply)*
 Shots are painful
 Risk of complications (e.g., bleeding, infection)
 Frequent visits to see doctor/nurse
 Transportation/parking costs
 Cost to the health care system
 Other: ___________________________________

24. What do you think would be the disadvantages of taking B_{12} in the form of pills? *(select all that apply)*
 I take too many pills already
 I would have to pay for them
 I won't get to see my doctor/nurse as often
 They won't work as well as the injections

☐ Other: ___________________________________

25. Would you be willing to participate in a 30-minute interview to tell us more about how you feel about B_{12} injections and pills?
☐ Yes ☐ No

26. If we provided you with a six (6) month supply of B_{12} pills free of cost, would you be willing to switch from B_{12} injections to B_{12} pills?
☐ Yes ☐ No

27. Please use the space below to share any other comments you may have:
