# Supplementary material for: Oral vitamin B12 therapy in the primary care setting: a qualitative and quantitative study of patient perspectives
Source: BMC Fam Pract. 2005 Feb 21;6:8. doi: 10.1186/1471-2296-6-8 (PMC554115; doi:10.1186/1471-2296-6-8)
Supplement: Additional File 2 — Follow-up questionnaire [file 1471-2296-6-8-S2.pdf]

Follow-up questionnaire

1. Name: ___________________________

2. Personal perception of health:
 Poor Below average Average Above average Excellent

3. On average, how many times in a month did you forget to take your B_{12}?
 0 1-2 3-5 6-9 10+

4. While you were taking the B_{12} pills, how many times on average did you see your doctor each month?
 0 1 2 3-4 5+

5. Compared to the injections, B_{12} pills made me feel:
 Much worse Worse The same Better Much better
 Don't know

6. How satisfied were you with the B_{12} pills?
 Very unsatisfied Unsatisfied Neutral Satisfied Very satisfied

7. What do you feel were the disadvantages of getting B_{12} by injection? *(select all that apply)*
 Shots are painful
 Risk of complications (e.g., bleeding, infection)
 Frequent visits to see doctor/nurse
 Transportation/parking costs
 Cost to the health care system
 Other: ___________________________________

8. What do you think were the disadvantages of taking B_{12} in the form of pills? *(select all that apply)*
 I take too many pills already
 I would have to pay for them
 I won't get to see my doctor/nurse as often
 They won't work as well as the injections
 Other: ___________________________________

9. Now that you have tried both injections and pills, which would you choose?
 I would go back to injections I would continue on pills

10. Please use the space below to share any other comments you may have:
