# Supplementary material for: Oral vitamin B12 therapy in the primary care setting: a qualitative and quantitative study of patient perspectives
Source: BMC Fam Pract. 2005 Feb 21;6:8. doi: 10.1186/1471-2296-6-8 (PMC554115; doi:10.1186/1471-2296-6-8)
Supplement: Additional File 3 — Reasons for switching to oral therapy [file 1471-2296-6-8-S3.pdf]

Reasons for switching to oral therapy

Convenience and time savings

"It would save me that hassle of coming in. It's like going into the Internet to do my banking rather than driving down to the bank. It's the whole time factor."

"It was something I could take every day and I didn't have to mark it down on my calendar, or remember to have to come in to take a B_{12} shot. Not always is it convenient to come in… you go to the office, and it could be very busy, and I'd come and I would park, and I would come in and… I'd say, 'Can you give me a quick shot?' and they'd usually find a time to squeeze me in, but at times, it was too busy, and sometimes I might have to wait an hour to have a shot, and granted, I accepted that if there was no other method of treatment, but certainly having the pills every morning with breakfast was far easier on a day-to-day basis."

Savings to health care system

"I guess the other issue is a bit of a social conscience, saying the fact that if it's time for me, it's also time for the staff, and they're taking time out of their day to do something for me, where in fact, if I can do it on my own, that time could be left for somebody else. We're all aware of the crunch on hospitals and time and particularly experienced nurses… why am I taking up their time?"

"I'm very supportive of saving the government some money, and I think everybody's a winner."

Ease of travel

"Well, if I go away I would be taking them with me, whereas it might be a little awkward to get shots."

Non-compliance with injections

"…when I went [in]to business, it was kind of a pain to get here, and I wasn't as – to be quite honest, I didn't take them as often as I should have. In other words, if I was busy at the office, instead of being here at the end of each month, I might drag it out to six weeks. That, probably, health-wise, wasn't the best thing."

Complications of injections

"I'm worried about the injection. I have a sister who's an RN, and one of the girls that I work with in the volunteer work say that you should never do it the muscle in the arm for B_{12}. They said I'm too thin, and it really hurts. Also, my sister said that if they do it the wrong way, they can paralyze you. I think she's jumping to extremes there, but they say it's much better to do it in the thigh. But here, they don't do it; they just do it up here [in the deltoid]."

Interest in research study

"I believe in research, and I think that this was obviously done for a purpose, and it was done for a positive purpose, so therefore, I didn't see any reason not to switch for the time being."
