# Supplementary material for: Oral vitamin B12 therapy in the primary care setting: a qualitative and quantitative study of patient perspectives
Source: BMC Fam Pract. 2005 Feb 21;6:8. doi: 10.1186/1471-2296-6-8 (PMC554115; doi:10.1186/1471-2296-6-8)
Supplement: Additional File 4 — Reasons for not switching to oral therapy [file 1471-2296-6-8-S4.pdf]

Reasons for not switching to oral therapy

Polypharmacy

"It doesn't appeal to me, taking the pill. I already take blood pressure pills because I have high blood pressure, and I take vitamin pills, and recently another doctor's given me something for my digestion, my esophagus. That's quite a bit of pills to go in your system."

Oral medications

"Well, the main reason is that I try not to put anything in me. …I never take pills unless I have to..."

Fear of side effects

"…I get bad reactions to pills, so I guess I'm just scared to take one more."

Concern about swallowing difficulties; forgetting to take daily medication

"Just having it done and over with, and not having to worry about choking, not having to remember to take it every day."

Loss of contact with health care providers and opportunities for drop-in consultations

"I mean, I won't be over here as often, and I won't see… the nurses – you know, sometimes it's always nice to talk to them, because it's much more difficult to get in to see the doctor, so you can say, 'What do you think about this?'"

Injections work faster and are more direct

"…somebody said that B_{12} works quicker if it goes directly into your bloodstream. That's what I heard."
